# Osteitis of the cuboid on unknown foreign body

**DOI:** 10.11604/pamj.2018.29.29.9764

**Published:** 2018-01-12

**Authors:** Hassane Zejjari

**Affiliations:** 1 Department of Trauma and Orthopedic Surgery, Military Hospital Moulay Ismail, Meknès, Morroco

**Keywords:** Osteitis, foreign body, cuboid

## Image in medicine

This is a patient aged 35 years old, farmer, who consults for chronic fistula of the soles lasting for three months without any notion of trauma. The review found a swelling of the outer edge of the foot of inflammatory appearance with pus coming through a fistula at the top. Plain radiography shows a well limited round osteolysis recalling the appearance of an abscess of Broca. The scanner confirms the osteolysis and highlights intra-osseous foreign body. The patient underwent surgical treatment by trimming, removal of a piece of wood and curettage of the bone cavity. The outcome was favorable with disappearance of infectious signs and filling the bone cavity.

**Figure 1:**
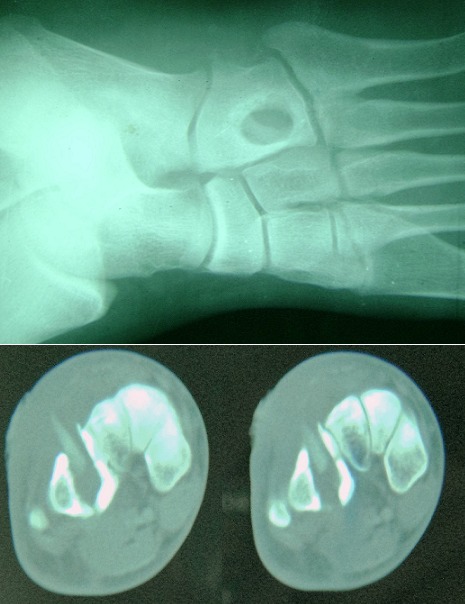
standard radiograph of the foot and two CT scans that objective osteolysis and the foreign body intraosseous the cuboid

